# Zirconia phase transformation in retrieved, wear simulated, and artificially aged ceramic femoral heads

**DOI:** 10.1002/jor.23589

**Published:** 2017-06-20

**Authors:** Maria Parkes, Kathryn Sayer, Markus Goldhofer, Philippa Cann, William L. Walter, Jonathan Jeffers

**Affiliations:** ^1^ Department of Mechanical Engineering Imperial College London London SW7 2AZ United Kingdom; ^2^ Mater Hospital Sydney Australia

**Keywords:** Alumina Toughened Zirconia, hip arthroplasty, Raman spectroscopy, phase transformation, implant wear

## Abstract

Zirconia in Zirconia toughened alumina ceramic hip replacements exists in an unstable state and can transform in response to stress giving the material improved fracture toughness. Phase transformation also occurs under hydrothermal conditions such as exist in vivo. To predict the hydrothermal aging that will occur in vivo accelerated aging procedures have been used, but validation of these models requires the study of retrieved hip joints. Here 26 retrievals are analysed to determine the degree of phase transformation in vivo. These were compared with virgin heads, heads that had undergone the accelerated aging process and heads wear tested to 5 million cycles in a hip simulator. Monoclinic content and surface roughness were measured using Raman spectroscopy and white light interferometry respectively. The monoclinic content for retrieved heads was 28.5% ± 7.8, greater than twice that in virgin, aged, or wear tested heads and did not have a significant correlation with time, contrary to the predictions of the hydrothermal aging model. The surface roughness for retrieved heads in the unworn area was not significantly different to that in virgin, aged, or unworn areas of wear tested heads. However in worn areas of the retrieved heads, the surface roughness was higher than observed in wear simulator testing. These results indicate that current testing methodologies do not fully capture the operational conditions of the material and the real performance of future new materials may not be adequately predicted by current pre‐clinical testing methods. © 2017 The Authors. *Journal of Orthopaedic Research* Published by Wiley Periodicals, Inc. on behalf of Orthopaedic Research Society 35:2781–2789, 2017.

Zirconia toughened alumina (ZTA) composite ceramics aim to combine the fracture toughness of zirconia and the wear resistance and chemical stability of alumina.^1^ One such ceramic is Biolox^®^ *delta* which, since its introduction in 2000, has been used in over 600,000 total hip replacements.^2^ This material has shown improvements over previous ceramics such as Biolox^®^ *forte* in both reduced numbers of fractures^3^ and reduced wear volumes.^4^ The improved performance is due to nano‐sized particles of yittria‐stabilized tetragonal zirconia polycrystals (Y‐TZP) distributed through the alumina matrix which improves the materials fracture toughness. The tetragonal zirconica can undergo a transformation to a monoclinic phase along with a volume increase of between 3% and 4% that induces compressive stresses and inhibits crack propagation.^5^ Although generally beneficial this transformation can also be triggered by a moist environment such as that found in the hip, in a process known as low temperature degradation (LTD).^5^ Such degradation for pure zirconia ceramics has been shown to reduce fracture toughness^6^ and lead to surface uplifts up to 200 nm in height^7^ which can increase wear. It is therefore important to understand this process for Alumina/Zirconia composite ceramics, including the rate at which they occur and the effect they have on the component.

The current methodology to predict the phase transformation that will occur in vivo is based on the assumption that the growth of monoclinic nuclei is thermally activated.^8^ Chevalier et al.^7^ and Pezzotti et al.^9^ have conducted a series of tests measuring the transformed monoclinic fraction as a function of time at elevated temperatures. From these results, they have determined an activation energy for ZTA through application of the Mehl–Avrami–Johnson (MAJ) theory.^10^ This activation energy has then been used to extrapolate the results to the relatively low body temperature and allows for estimation of monoclinic fraction with implantation time in vivo. Based on the reported activation energy in^8^ an accelerated aging protocol, that would simulate the in vivo conditions, was suggested in which 1 h of accelerated aging for ZTA is expected to be equivalent to 3.9 years in vivo.^1^, ^8^ This protocol, adopted by the ISO standard 6474‐2:2012,^11^ is to autoclave the ceramic at 134°C, under two bars water steam, for 10 h to simulate 39 years of in vivo conditions.

Validating this model requires retrieval analysis. Such a comparison by Arita et al.^12^ demonstrated that the ISO standard for accelerated aging underestimated the in vivo phase transformation for pure zirconia heads. A limited number of retrieval studies exist for the ZTA composites as summarized in Table 1, with results for only 42 femoral heads. These studies typically show a monoclinic content of 25–33% after average follow up times of between 2 and 5 years. Using the MAJ model 2–5 years would be simulated by approximately 1 h of aging at 134°C, under two bars water steam, but this would only generate an average monoclinic content of 15%.^8^ The retrieval results in Table 1 are higher than this and closer to the 23% ± 5 found by Chevalier et al.^8^ for heads undergoing the ISO standard aging procedure, which should have simulated 39 years in vivo. However, there may be some discrepancy due to the difference in measurement techniques as both Raman spectroscopy and X‐ray diffraction (XRD) are used by different authors^13^, ^14^, ^15^ to assess monoclinic content.

**Table 1:** Retrieval Studies on Biolox^®^ Delta

Study	Number of Retrievals With Monoclinic Content Reported	Average Follow Up (Years)	Measurement Technique	Average Monoclinic Content	Control Sample Monoclinic Content
Elpers et al.^14^	27	2	Raman	25.3% ± 8.3	None
Taddei et al.^15^	6	3.2	Raman	25.5% ± 12.8	11% ± 2
Affatato et al.^16^	2	4.6	Raman	33%	None
Clarke et al.^13^	1	3	XRD	9.2%	14%
Zhu et al.^17^	2	3.5	Raman	34.2%	20.4% ± 6.0
Perrichon et al.^18^	4	Not given	Raman	35% ± 7	10%

Previous retrieval studies on ZTA ceramics also disagree on the contribution of wear to an increase in monoclinic phase.^4^, ^14^, ^15^, ^19^ It is generally agreed that severe wear, such as caused by edge loading and microseparation does increase monoclinic content in the worn areas.^4^, ^13^, ^19^ Recent studies also show metal contamination of the surface which results in wear of the ceramic can also increase monoclinic content.^16^, ^17^ Sources of metal contamination include contact with a metallic articulating surface, dislocation, or impingement leading to contact with the acetabular rim and dislocation/relocation manoeuvres during surgery.^16^ However, results from well‐functioning implants are not so conclusive. Elpers et al.^14^ reported no significant increase in monoclinic content due to mild wear, but Taddei et al.^15^ reported wear to be the main cause of phase transformation.

The aim of this study is therefore to investigate further the extent to which artificial aging of ZTA ceramic parts represents the clinical scenario. Further to this, we will also investigate how long term pre‐clinical testing (wear simulator tests) influences the monoclinic transformation of the material, and compare these to the clinical scenario. These data will be important for the orthopedic community to have greater understanding on how this material behaves both in vivo and in the laboratory testing that aims to simulate the in vivo environment.

## EXPERIMENTAL PROCEDURE

### Materials

Twenty‐six retrieved ZTA femoral heads made from Biolox^®^ *delta* (CeramTec AG, Plochingen, Germany) were included in the study. These were compared with five virgin, five artificially aged, and six wear simulated femoral head resurfacing components made from Biolox^®^ *delta* (CeramTec AG, Plochingen, Germany) manufactured in 2015.

The retrievals were obtained after implantation of between 1 month and 7 years with an average follow‐up of 1.5 years. All retrievals were from ceramic‐on‐ceramic hip joints that were implanted between 2004 and 2012. Mean patient age was 69 years (range 48–87 years). Patient demographic data is given in Table 2.

**Table 2:** Patient Demographic Data for the 26 Retrieved Biolox^®^ Delta Components

ID#	Days Implanted	Age	Gender	Side	Implantation Year	Reason for Revision
6744	133	78	Female	Right	2006	Dislocation
8965	646	56	Female	Left	2010	Leg length discrepancy/pain
6469	28	87	Female	Left	2006	Dislocation/subsidence
9125A	403	65	Male	Right	2011	Periprosthetic fracture
7722	355	79	Male	Right	2009	Recurrent dislocation
6490	23	87	Male	Left	2006	Unstable hip
8548	1035	82	Female	Right	2008	Loose acetabular component
6746	69	81	Female	Left	2007	Recurrent dislocation
5764	216	61	Female	Right	2004	Dislocation
8786	1951	72	Female	Right	2006	Acetabular fracture and loosening
8658	90	48	Female	Left	2011	Dislocation
8405	703	64	Male	Right	2009	Wear
9423	877	75	Male	Right	2011	Non union of femoral fracture with failure of fixation
6733	91	56	Female	Right	2007	Deep infection
7393	91	79	Female	Right	2008	Loose acetabular component/osteolysis
9403	1253	72	Female	Left	2010	Loosening femoral component
9125B	403	65	Male	Right	2011	Periprosthetic fracture
7734	28	73	Female	Left	2009	Periprosthetic fracture
7277	34	57	Female	Right	2008	Subluxation
9816	334	65	Female	Left	2012	Failed acetabular cage
9514	2478	76	Male	Right	2007	Periprosthetic fracture
8749	931	67	Female	Right	2009	Cup has not ingrown after revision
8263	1001	67	Female	Left	2007	Recurrent subluxation
9026	405	58	Female	Left	2011	Clunking/large effusion
8580	539	62	Female	Left	2009	Infection
6300	62	61	Male	Right	2006	Wear

The five virgin femoral head resurfacing components were measured for surface roughness and monoclinic content (as described below) and then cumulatively aged for 1, 2, 5, and 10 h in a SanoClav autoclave (Adolf Wolf SanoClav, Bad Uberkingen, Germany) under vapor at two bar pressure and at 134°C. These conditions are in‐line with ISO 6474‐2:2012.^11^ Repeat surface roughness and monoclinic measurements were made after each aging period.

Six femoral head resurfacing components were tested for five million cycles in accordance with ISO 14242‐1:2014.^20^ Wear tests were conducted on a six station hip simulator at 1 Hz, and 37 ± 2°C. These samples are referred to as wear simulated. Surface finish and monoclinic measurements were made (as described below) after the completion of wear simulation.

### Monoclinic Content Measurement

**Figure 1:**
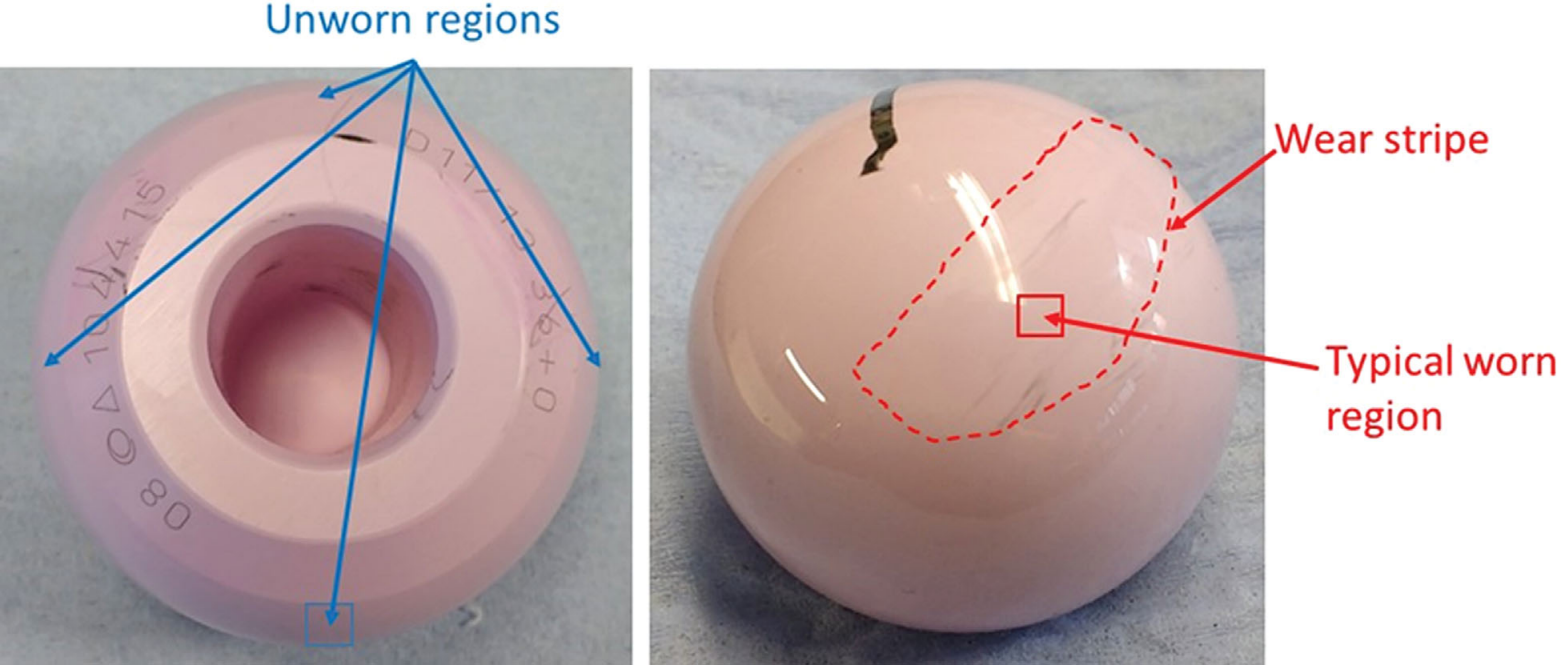
Retrieved Biolox^®^ *delta* head showing typical locations of Raman measurement areas.

Monoclinic content was measured using Raman Spectroscopy. For the retrievals, measurements were made on each component at four equi‐spaced locations about the axis of the head, on the bearing surface and close to the trunnion hole, to capture the unworn surface (Fig. 1a). The monoclinic content was also measured in the center of the worn area (which was detected in 15 of the 26 retrieval components) to represent the worn area as shown in Figure 1b. For the wear simulated components, monoclinic content was measured in the worn and unworn area. For the virgin and aged components, there was no worn area, so monoclinic content was only measured in the unworn area.

Raman spectra were collected using an Alpha300R, confocal Raman spectrometer (WITec Gmbh, Ulm, Germany) using a laser wavelength of 532 nm, a 50× objective and a 100 µm pinhole. Two thousand five hundred spectra were recorded over an area of 10 × 10 μm. Cosmic Ray removal, background correction, and averaging of the 2500 points to give one spectra were conducted using Witec Project Software. Peak fitting using a Lorentz peak fitting function was applied in Origin Pro to give the relative peak intensities of the tetragonal (Im_145_ and Im_260_) and monoclinic (Im_178_ and Im_189_) peaks. The average monoclinic content was calculated based on the method of Clarke and Adar^21^ following the formula:

(1) $$V_{m}=\frac{I_{m}^{178}+I_{m}^{189}}{0.97(I_{t}^{145}+I_{t}^{260})+I_{m}^{178}+I_{m}^{189}}$$

and reported as average ± standard deviation.

### Surface Roughness Measurement

Surface roughness was measured by White light interferometry using a Wyko NT9100 Optical Profiling System with a 20× objective (Veeco Instruments Inc., NY) to study surface roughness at the macroscale. The average value for surface roughness, *R_a_* was calculated over an area of 430 × 570 µm and reported as average ± standard deviation.

### Statistical Analysis

Statistical analysis was performed in SPSS v 22 (IBM Corp), with significance level set at *p* < 0.05. For comparisons across all groups a one‐way ANOVA with post hoc Tukey HSD tests was used. For comparisons between heads independent samples *t*‐tests were used. The Pearson product‐moment correlation coefficient was used to assess the linear correlation between variables. For successive measurements on the aged heads a repeated measures ANOVA was used to compare means.

## RESULTS

### Virgin, Accelerated Aged, and Wear Simulated Heads

The five virgin ZTA components had a mean monoclinic content, *V*_m_, of 5.9% ± 1.4. The monoclinic content increased with hydrothermal aging time to 9.2% ± 1.5 at 1 h, 12.3% ± 0.7 at 2 h, 12.1% ± 2.8 at 5 h and 13.1% ± 3.6 at 10 h. Wear simulated heads were measured both in the unworn and worn area. The average *V*_m_ for the unworn area was 3.7% ± 1.1 while the average *V*_m_ in the worn area was 3.1% ± 0.4. A post hoc Tukey HSD found no statistically significant change in monoclinic content between the unworn and worn areas.

The mean value of surface roughness, *R*_a_ for virgin heads was 9.6 nm ± 0.7. With aging from 0 to 10 h there was a slight increase in the average *R_a_*, but this was not statistically significant [*F*(4,20) = 0.141, *p* = 0.965]. This indicates that increasing monoclinic content alone does not lead to increases in surface roughness. For wear simulated heads the mean value of *R_a_* in the unworn area was 10.3 nm ± 0.6 compared with 14.8 nm ± 2.7 in the worn area. This increase was in line with results from the literature which show increased surface roughness in worn areas.^4^, ^13^, ^17^, ^22^

### Retrievals

**Figure 2:**
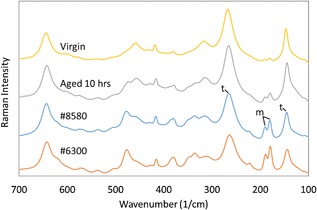
Average Raman spectra recorded on representative heads. From the top: Virgin head, aged head after 10 h autoclave at 134°C, two bar water, Unworn area Retrieval #8580 (1.5 years follow up), Unworn area retrieval #6300 (62 days follow up). The tetragonal (t) and monoclinic (m) bands used for analysis are indicated.

**Figure 3:**
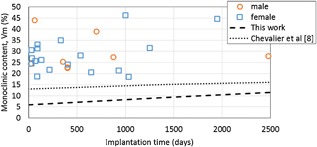
Monoclinic content as measured on unworn areas of retrievals. Dashed lines showing expected monoclinic content from artificial aging model based on the measurements in this study, and the hydrothermal aging results of Chevalier et al.^8^

**Figure 4:**
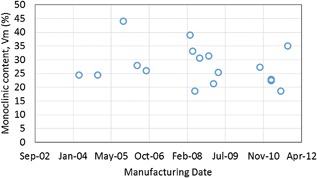
Monoclinic content as measured on unworn areas of retrievals showing that there is no correlation with manufacturing date.

**Figure 5:**
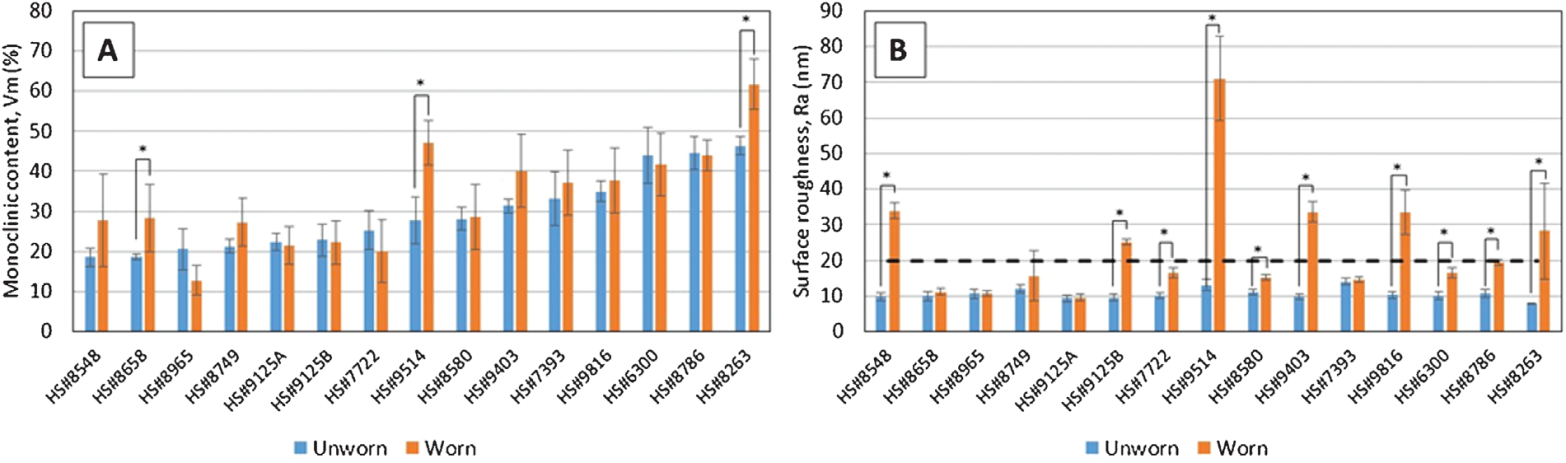
Comparison of unworn and worn regions for individual retrievals with visible wear scar (15 of 26) by (A) monoclinic content (B) surface roughness with dashed line showing limit of *R*_a_ specified by ISO 7206‐2:2011^23^ for ceramic hip joint prostheses before implantation.

All retrievals showed distinct Raman peaks for the monoclinic phase at 178 and 189 cm^−1^ as shown in Figure 2. The average monoclinic content of unworn areas of all the retrieved heads was 28.5% ± 7.8 at an average 1.5 years follow up, which was higher than expected for short term retrievals. This is shown in Figure 3 where all points lie above the line calculated from the hydrothermal aging results of this study and the results of Chevalier et al.^8^ on which the ISO standard is based. It should be highlighted that we cannot know the starting monoclinic content when the retrievals were implanted which may have been higher than those heads used for these artificial aging models, but Table 1 provides some previously reported data. No significant correlation between implantation time and *V*_m_ content was found (Pearson product‐moment correlation, *r* = 0.234, *n* = 26, *p* = 0.250). The mean monoclinic content of the retrievals <1 month was 26.5% ± 3.5 (*n* = 3) and >1 and <3 months in situ was 29.9% ± 8.6 (*n* = 6) which was not statistically different from that of retrievals >3 months at 28.4% ± 8.3 (*n* = 17) (Fig. 3) [*F*(2,23) = 0.191, *p* = 0.827]. There was also no statistically significant difference in *V*_m_ between female–male (independent samples *t*‐test, *p* = 0.791) or right–left groups (independent samples *t*‐test, *p* = 0.682). There was also no correlation between time since manufacture and *V*_m_ content (Pearson product‐moment correlation, *α* = 0.05, *p* = 0.445) as can be seen in Figure 4. For 12 out of 15 retrievals with visible wear scars, the *V*_m_ content of worn areas was not statistically different to the unworn areas (Fig. 5) agreeing with the data from the wear simulated resurfacing components. For 3 out of 15 retrievals, significant differences were found (independent samples *t*‐test, *p* = 0.009, *p* < 0.001 and *p* <0.001 for #8658, #9514 and #8263 respectively).

**Figure 6:**
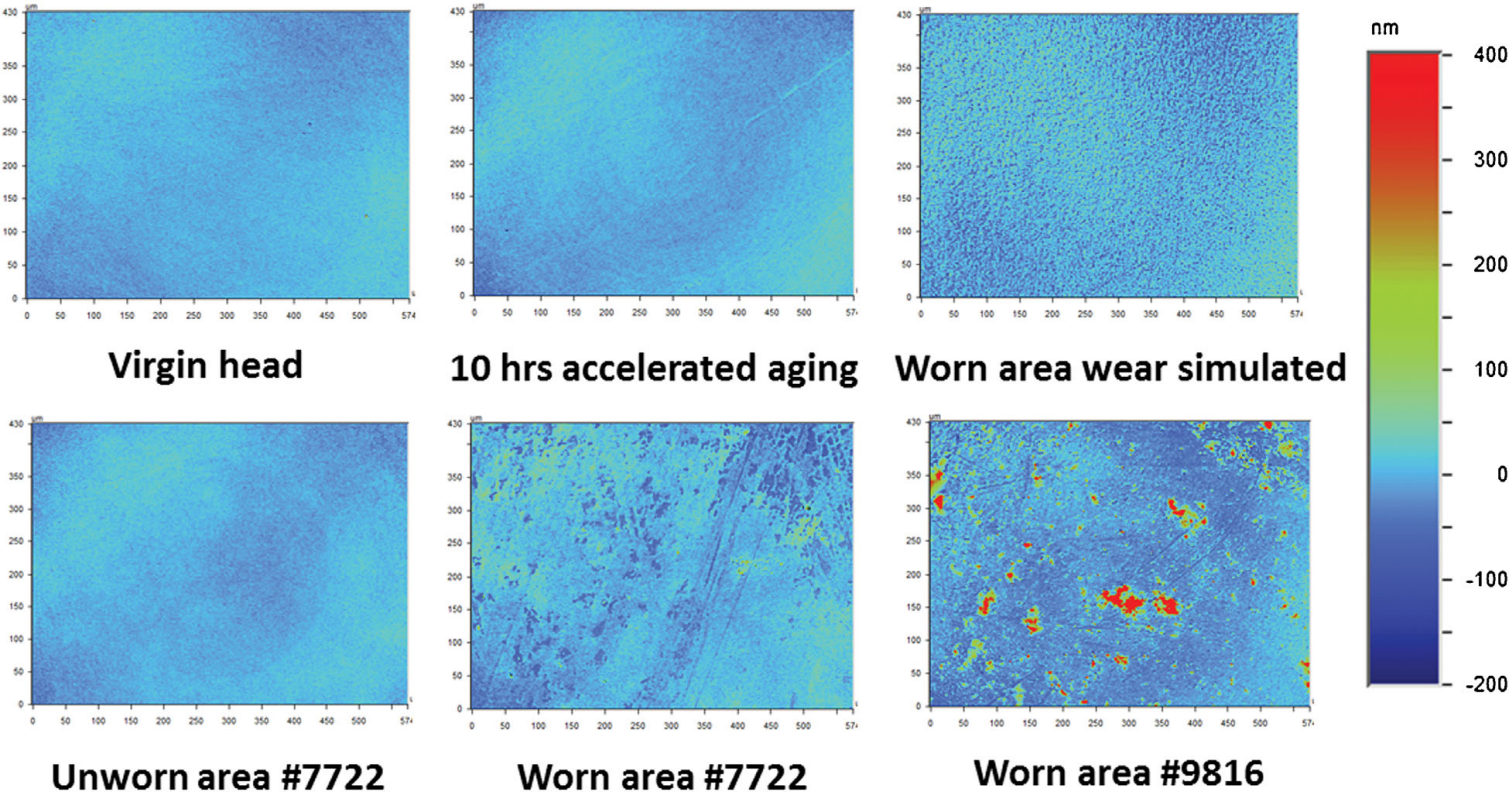
Typical surface roughness maps, *R*_a_ as measured by white light interferometry for all types of femoral heads showing increased surface roughness and features in wear regions.

**Figure 7:**
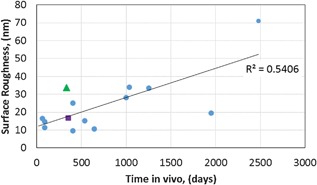
Surface roughness in worn area as measured on retrieved femoral heads as a function of time. Retrievals #7722 (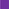) and #9816 (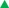) from Figure 5 are highlighted.

In the unworn areas of the 26 retrievals, the mean value of *R*_a_ was 11.7 ± 3.8 nm (Fig. 5b). Of the 26 retrieved samples, only 15 had visible areas of wear (Fig. 6). In these worn areas, the mean *R*_a_ increased to 24.2 ± 16 nm, and reached a maximum of 70.9 nm (Fig. 4b). The increase in surface roughness in the worn area was correlated with implantation time (Pearson product‐moment correlation, *r* = 0.718, *n* = 15, *p* = 0.003) as shown in Figure 7. The increase in surface roughness did not correlate with the monoclinic content suggesting that the measured wear was not sufficient to induce phase transformation.

### Comparison Across Groups

**Figure 8:**
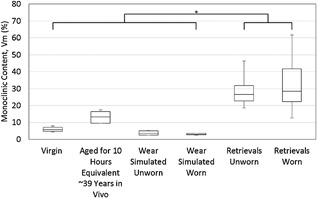
Comparison of Monoclinic content across all measured groups. A significant difference was found in *V*_m_ at *p* < 0.05 level [*F*(5,57) = 26.4, *p* < 0.001] with post hoc comparisons using the Tukey HSD finding that the average monoclinic content in the unworn area of retrievals is significantly different than either the virgin, artificially aged, or wear simulated samples though not significantly different from the worn area of the retrievals.

The average monoclinic content for all samples is shown in Figure 8. The average monoclinic content in the unworn area of retrievals was significantly different than either the virgin, artificially aged, or wear simulated samples though not significantly different from the worn area of the retrievals. We cannot be sure of the starting Vm of the retrievals, but Table 1 provides some previously reported data.

**Figure 9:**
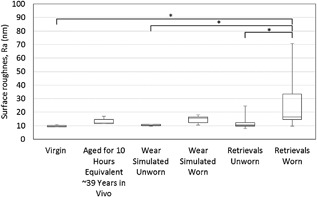
Comparison of Surface roughness across all groups. A significant difference in surface roughness [*F*(5,57) = 5.05, *p* = 0.001] was found. In contrast to the average *V*_m_, the post hoc tests showed the significant difference to be between the *R*_a_ of the worn area of the retrievals and the virgin and aged samples with no difference between the unworn area of retrievals and the surface roughness of the virgin, artificially aged, or wear simulated heads being observed.

The same comparison across groups for surface roughness is shown in Figure 9. The surface roughness in the unworn area of the retrievals was not significantly different to that of the virgin, wear simulated, or artificially aged parts. However, the surface roughness of the worn area of the retrievals was significantly different to the unworn area of the retrievals, the virgin, wear simulated, and artificially aged parts.

## DISCUSSION

Our work demonstrates that a greater level of t‐m transformation may be present in ZTA heads in vivo at an earlier implantation time than predicted by current hydrothermal aging models. This extra transformation is not due to mechanical energy from wear and as yet remains unexplained. These data suggest the current methods of artificially aging ZTA specimens do not fully replicate the clinical scenario. Our work also indicates that while mild wear and stripe wear roughen the surface of the ceramic in vivo, this does not always correlate with an increase in monoclinic transformation. This may mean that under normal operating conditions, which includes stripe wear from edge loading, the wear is not severe enough to induce phase transformation.

In this study, we have found an average *V*_m_ content in the unworn regions of all retrieved heads of 28.5% ± 7.8 at an average 1.5 years follow up. This is comparable to previous retrieval studies as reported in Table 1 that found values between 25% and 34%. This average monoclinic content is equivalent to or greater than that reported for heads artificially aged for 10 h at 134°C according to ISO 6474‐2:2012^11^ (13.1% ± 3.6 measured here or the 23% ± 5 reported by Chevalier et al.^8^). The current hydrothermal models predict the monoclinic content increase following ISO standard accelerated aging is equivalent to 40 years in vivo, whereas retrieval results are showing greater monoclinic content after less than a tenth of this time. In their recent paper, Zhu et al.^17^ suggested that chemistry driven processes due to the diffusion of metal ions into the ceramic lattice could induce what they termed as abnormally high levels of phase transformation in ceramic‐on‐metal THR. Our results and those from literature suggest that the levels of monoclinic transformation Zhu et al. report are not abnormally high but typical for short term retrievals and occur for ceramic on ceramic as well as for ceramic‐on‐metal THRs. The generally accepted model of LTD is based on the diffusion of OH^‐^ ions into the zirconia lattice to fill oxygen vacancies which acts to destabilize the tetragonal phase, although other ions such as O^2−^ and H^+^ have also been suggested to have a role.^5^ The presence of salts and proteins in the post‐operative joint space as well as a non‐neutral pH^24^ make the ion content quite different to the steam autoclave artificial aging environment. This may be a contributing factor in the discrepancy between in vivo data and artificial aging data.

In both our study and in^14^ some retrievals with monoclinic content higher than 30% were seen after less than 6 months in vivo suggesting a large increase in monoclinic content compared to virgin parts is either present at time of implantation or occurs almost immediately after implantation. If the monoclinic content continues to increase above this due to hydrothermal aging with time or reaches an upper bound^8^, ^13^ is not clear. In our study with 26 retrievals, we found no correlation between implantation time and monoclinic content (Fig. 3). This is in contrast to Elpers et al.^14^ who found a significant correlation between *V*_m_ and implantation time. A common limitation is that the studies have very limited number of longer term retrievals, with *n* = 6 in the Elpers study and *n* = 3 in this study being implanted for greater than 3 years. Longer term retrieval studies are therefore required to determine whether surface monoclinic content increases with time in vivo.

Our virgin samples had a low monoclinic content of 5.9% ± 1.4, which is lower than that reported by Chevalier et al.^8^ and also lower than reported for virgin heads by other authors in Table 1 and indicates that there are significant variations in initial monoclinic content from batch to batch. This could be an inherent aspect of the sintering process used to manufacture the material. As our virgin samples were manufactured in 2015 this may also indicate an improvement in initial monoclinic content with manufacturing date. If a reduced initial monoclinic content is due to material improvements that result in changes in the local environment of the zirconia crystals then this change may also impact the kinetics of phase transformation.^8^ This potential improvement has previously been discussed by Taddei et al.^15^ and Affatato et al.^22^ who found that in their studies of six retrievals Vm for heads manufactured in 2009 and later stayed low at around 10%. Although we did not find this (as shown in Fig. 4), it may be that batch to batch variability masked any improvement in more recently manufactured parts.

Our study indicated that wear simulator parts did not replicate the increased surface roughness, nor the level of monoclinic transformation observed in retrievals. A more severe wear simulator test is available where the head is pulled slightly out of the socket during the swing phase of gait, such that it crashes into the superior rim of the socket as it relocates when the load is applied for the next cycle. This is often termed microseparation wear simulation, and has been demonstrated to produce a stripe on the wear simulated components with a rougher surface finish^13^, ^25^ closer to our retrieval results. However analysis of this set of retrievals found four in five of the visible wear stripes were due to a posterior edge loading mechanism that occurs during deep flexion. Our finding is consistent with previous retrieval studies indicating posterior edge loading is the most common mechanism of stripe wear accounting for around 70% of instances.^26^ Posterior edge loading is a different mechanism to microseparation producing a stripe in a different location and orientation,^27^ and no wear simulator protocol is currently able to simulate this mechanism.

In this study, we did not find a correlation between phase transformation and increases in surface roughness indicative of wear on wear simulated heads, or 13 of 15 retrievals with visually identified worn areas. Monoclinic transformation has been shown to occur with application of stress to zirconia ceramics^21^ with transformation occurring above a critical local stress^5^ which has been reported to be around 300–400 MPa.^4^, ^9^, ^28^ In their work on wear of ZTA, Ma and Rainforth^19^, ^29^ found monoclinic transformation could be induced by sliding. Their tests had initial contact pressures in the region of 3,000 MPa due to the testing geometry, well above the critical local stress. Similarly increased phase transformation has been reported in microseparation wear tests^30^ where stresses have been predicted to increase to between 200 and 470 MPa depending on separation distance.^31^, ^32^ For our wear simulated heads where standard load cycles were applied contact stresses are predicted to be around 70 MPa^31^ which is below the critical local stress and so does not result in phase transformation. For the retrieved heads, three cases (#8658, #9514, and #8263) showed a statistically significant increase in *V*_m_ in the worn compared with the unworn area (Fig. 5). Two of these heads also showed a large increase in *R*_a_. Taken altogether, these results suggest that monoclinic transformation does occur as a result of wear but only above a certain threshold and the process is not well described by a simple correlation or simulated by standard gait‐cycle wear tests.

The increase in *R*_a_ with time observed here indicates the wear mechanism of the ZTA ceramic may be different to that reported for alumina ceramic where repolishing has been reported.^27^ Increases in ZTA roughness of retrieved implants compared with virgin materials have previously been reported^14^, ^16^ and are associated with both wear and metal transfer. However the relationship between *R*_a_ and time has not been tested by other authors and our understanding would benefit from further retrieval studies.

Limitations of this study include that we do not know with certainty the monoclinic content of the retrievals before they were used. The higher than expected level of phase transformation may have been present at implantation. If this was so, the implants would have been outside the manufacturer's general specification^1^ and have had higher monoclinic fractions than previously reported controls (Table 1). A further limitation is the number of retrievals and the short time for which they have been implanted, meaning our study cannot elucidate on what happens to monoclinic content in the long term. This is unavoidable as ZTA is a relatively new material in orthopedic surgery, demonstrated by the longest published follow up study by Hamilton et al.^33^ having a mean follow up of only 5 years. It also has a low revision rate, reporting only nine revisions in their study of 345 hips. In this study, we only measured monoclinic content at discrete locations on the femoral heads. Mapping the whole head would be unfeasible in terms of time to acquire and process the data, and may not have provided any further detail—when we measured the unworn surface of the retrievals, the variation was low in line with,^14^ and we also performed a pilot study on virgin parts that found low variation over the whole surface of the head. We also tested wear simulator parts under conventional conditions, without severe loading like microseparation which would have generated a more severe damage to the parts as has been discussed previously. A further limitation is that the resurfacing components used as control components are a different design to standard femoral heads. However, the material and bearing surface are prepared using the same manufacturing methods and by the same manufacturer.

Although we found that the hydrothermal model may not adequately predict levels of phase transformation observed in vivo, there is no evidence that the levels observed in vivo detrimentally affect the performance of ZTA. Indeed, we found in agreement with^8^ that phase transformation alone does not increase surface roughness, and also in the majority of cases, there was no additional phase transformation in worn areas compared to unworn areas of the head. Furthermore, phase transformation as high as 47% due to aging and fatigue testing has been shown to have no detrimental effect on the residual strength of the ZTA ceramic,^34^ while aging to around 70% *V*_m_ has not reduced the material bulk strength.^35^ Although these data can only elucidate on the short to mid term performance of the material, they indicate that the transformation toughening mechanism of ZTA does not cause problems at the unconstrained bearing surface in clinical practice, which matches the good clinical results that are reported for the material.^33^ However, the data presented here indicates pre‐clinical testing methods, currently being adopted as part of new product development by the orthopedic industry, do not adequately represent the clinical environment. While the 5‐year clinical follow‐up data indicate this has not been a problem for the specific ZTA material studied in this paper, it may be a cause for concern that newer ZTA materials could get released onto the market without full understanding of how they will perform in the in vivo environment.

## CONCLUSION

This study found that phase transformation of retrieved ceramic femoral heads exceeds that predicted in preclinical testing. In 80% of cases, this phase transformation was not triggered by wear of the bearing surface and points to a possible effect of the chemical nature of the in vivo environment. Although the phase transformation was greater than expected, there was no evidence of any detrimental effect for the ceramic material studied. Current preclinical testing may not fully capture the in vivo environment and this must be considered a risk when developing new materials for implantable devices.

## AUTHORS' CONTRIBUTIONS

MP and KS acquired and analysed the data and drafted and critically revised the paper. MG acquired and interpreted data and critically revised the paper. PC, WLW, and JJ contributed to the study design, interpretation of the data and critically revised the paper. All authors approved the submitted manuscript.

## References

[1] Kuntz M , Masson B , Pandorf T . 2009 Current state of the art of the ceramic composite material BIOLOX^®^ delta. Strength Mater 1:133–156.

[2] Jeffers JRT , Walter WL . 2012 Ceramic‐on‐ceramic bearings in hip arthroplasty: state of the art and the future. J Bone Jt Surg—Br 94:735–745. 10.1302/0301-620X.94B6.28801 22628586

[3] Massin P , Lopes R , Masson B , et al. 2014 Does Biolox^®^ Delta ceramic reduce the rate of component fractures in total hip replacement? Orthop Traumatol Surg Res 100:S317–S321. 25130763 10.1016/j.otsr.2014.05.010

[4] Clarke IC , Pezzotti G , Green DD , et al. 2005 Severe Simulation Test for run‐in wear of all‐alumina compared to alumina composite THR Bioceramics and alternative bearings in joint arthroplasty. Steinkopff: Darmstadt p 11–20.

[5] Chevalier J , Gremillard L , Virkar AV , et al. 2009 The tetragonal‐monoclinic transformation in zirconia: lessons learned and future trends. J Am Ceram Soc 92:1901–1920.

[6] Haraguchi K , Sugano N , Nishii T , et al. 2001 Phase transformation of a zirconia ceramic head after total hip arthroplasty. J Bone Joint Surg Br 83:996–1000. 11603539 10.1302/0301-620x.83b7.12122

[7] Chevalier J , Cales B , Drouin JM . 1999 Low‐temperature aging of Y‐TZP ceramics. J Am Ceram Soc 82:2150–2154.

[8] Chevalier J , Grandjean S , Kuntz M , et al. 2009 On the kinetics and impact of tetragonal to monoclinic transformation in an alumina/zirconia composite for arthroplasty applications. Biomaterials 30:5279–5282. 19577804 10.1016/j.biomaterials.2009.06.022

[9] Pezzotti G , Yamada K , Sakakura S , et al. 2008 Raman spectroscopic analysis of advanced ceramic composite for hip prosthesis. J Am Ceram Soc 91:1199–1206.

[10] Christian JW . 2002 The theory of transformations in metals and alloys. Oxford: Pergamon p 422–479.

[11] International Standards Organisation (ISO). ISO 6474–2 Implants for surgery—ceramic materials. Part 2: Composite materials based on a high purity alumina matrix with zirconia reinforcement 2012.

[12] Arita M , Takahashi Y , Pezzotti G . 2015 Environmental stability and residual stresses in zirconia femoral head for total hip arthroplasty: in vitro aging versus retrieval studies. Biomed Res Int 2015. Article ID 638502. 10.1155/2015/638502 PMC4469792 26146624

[13] Clarke IC , Green DD , Williams PA , et al. 2009 Hip‐simulator wear studies of an alumina‐matrix composite (AMC) ceramic compared to retrieval studies of AMC balls with 1‐7 years follow‐up. Wear 267:702–709.

[14] Elpers M , Nam D , Boydston‐White S , et al. 2014 Zirconia phase transformation, metal transfer, and surface roughness in retrieved ceramic composite femoral heads in total hip arthroplasty. J Arthroplasty 29:2219–2223. 25212282 10.1016/j.arth.2014.08.011

[15] Taddei P , Modena E , Traina F , et al. 2012 Raman and fluorescence investigations on retrieved Biolox delta femoral heads. J Raman Spectrosc 43:1868–1876.

[16] Affatato S , Ruggiero A , De Mattia JS , et al. 2016 Does metal transfer affect the tribological behaviour of femoral heads? Roughness and phase transformation analyses on retrieved zirconia and Biolox^®^ Delta composites. Compos Part B Eng 92:290–298.

[17] Zhu W , Pezzotti G , Boffelli M , et al. 2016 Chemistry‐driven structural alterations in short‐term retrieved ceramic‐on‐metal hip implants: evidence for in vivo incompatibility between ceramic and metal counterparts. J Biomed Mater Res Part B Appl Biomater 1–12. https://doi.org/10.1002/jbm.b.33689 10.1002/jbm.b.33689 27087384

[18] Perrichon A , Reynard B , Gremillard L , et al. 2015 Effects of in vitro shocks and hydrothermal degradation on wear of ceramic hip joints: towards better experimental simulation of in vivo ageing. Tribol Int 100:410–419.

[19] Ma L , Rainforth WM . 2010 A study of Biolox delta subject to water lubricated reciprocating wear. Tribol Int 43:1872–1881.

[20] International Standards Organisation (ISO). ISO 14242–1 Implants for surgery—wear of total hip‐joint prostheses. Part 1: Loading and displacement parameters for wear‐testing machines and corresponding environmental conditions for test 2014.

[21] Clarke D , Adar F . 1982 Measurement of the crystallographically transformed zone produced by fracture in ceramics containing tetragonal zirconia. J Am Ceram Soc 284–288.

[22] Affatato S , Modena E , Toni A , et al. 2012 Retrieval analysis of three generations of Biolox^®^ femoral heads: spectroscopic and SEM characterisation. J Mech Behav Biomed Mater 13:118–128. 22842282 10.1016/j.jmbbm.2012.04.003

[23] International Standards Organisation (ISO). ISO 7206–2:2011 Implants for surgery—partial and total hip joint prostheses. Part 2: Articulating surfaces made of metallic, ceramic and plastics materials 2011; 2011.

[24] Brandt J‐M , Brière LK , Marr J , et al. 2010 Biochemical comparisons of osteoarthritic human synovial fluid with calf sera used in knee simulator wear testing. J Biomed Mater Res A 94:961–971. 20730933 10.1002/jbm.a.32728

[25] Williams SR , Wu JJ , Unsworth A , et al. 2011 Wear and surface analysis of 38 mm ceramic‐on‐metal total hip replacements under standard and severe wear testing conditions. Proc Inst Mech Eng Part H J Eng Med 225:783–796. 10.1177/0954411911404773 21922955

[26] Esposito CI , Walter WL , Roques A , et al. 2012 Wear in alumina‐on‐alumina ceramic total hip replacements: a retrieval analysis of edge loading. J Bone Joint Surg Br 94:901–907. 22733943 10.1302/0301-620X.94B7.29115

[27] Walter WL , Insley GM , Walter WK , et al. 2004 Edge loading in third generation alumina ceramic‐on‐ceramic bearings: stripe wear. J Arthroplasty 19:402–413. 15188097 10.1016/j.arth.2003.09.018

[28] Sergo V , Clarke DR , Clake DR . 1995 Deformation bands in ceria‐stabilized tetragonal zirconia/alumina: II, stress‐induced aging at room temperature. J Am Ceram Soc 78:641–644.

[29] Ma L , Rainforth WM . 2012 The effect of lubrication on the friction and wear of Biolox^®^ delta. Acta Biomater 8:2348–2359. 22342830 10.1016/j.actbio.2011.12.037

[30] Clarke IC , Green D , Williams P , et al. 2006 US perspective on hip simulator wear testing of BIOLOX^®^ delta in’ severe’ test modes In: Benazzo F, Falez F, Dietrich M, editors. Bioceramics and alternative bearings in joint arthroplasty. 11th Biol. Symp. Rome, June 30–July 1, 2006 Proc. Steinkopff: Darmstadt p 189–205.

[31] Sariali E , Stewart T , Jin Z , et al. 2012 Effect of cup abduction angle and head lateral microseparation on contact stresses in ceramic‐on‐ceramic total hip arthroplasty. J Biomech 45:390–393. 22119582 10.1016/j.jbiomech.2011.10.033

[32] Mak MM , Jin ZM . 2002 Analysis of contact mechanics in ceramic‐on‐ceramic hip joint replacements. Proc Inst Mech Eng Part H J Eng Med 216:231–236. 10.1243/09544110260138718 12206519

[33] Hamilton WG , McAuley JP , Blumenfeld TJ , et al. 2015 Midterm results of delta ceramic‐on‐ceramic total hip arthroplasty. J Arthroplasty 30:110–115. 26122108 10.1016/j.arth.2015.02.047

[34] Kuntz M . 2007 Live‐time prediction of BIOLOX^®^ delta In: Chang J‐D, Billau K, editors. Bioceramics and Alternative Bearings in Joint Arthroplasty. 12th Biol. Symp. Seoul, Repub. Korea Sept. 7‐8, 2007 Proc. Steinkopff: Darmstadt p 281–288.

[35] Pezzotti G , Yamada K , Porporati AA , et al. 2009 Fracture toughness analysis of advanced ceramic composite for hip prosthesis. J Am Ceram Soc 92:1817–1822.

